# When Every Test Fails: Multifocal Grade 1 Ileal Neuroendocrine Tumors Causing Transfusion-Dependent Obscure Overt Gastrointestinal Bleeding

**DOI:** 10.14309/crj.0000000000002326

**Published:** 2026-10-21

**Authors:** James Mo, Suraj Suresh

**Affiliations:** 1 Department of Medicine, Henry Ford Hospital, Detroit, MI; 2 Division of Gastroenterology and Hepatology, Henry Ford Health, Detroit, MI

**Keywords:** small bowel neuroendocrine tumor, obscure overt gastrointestinal bleeding, exploratory laparotomy

## Abstract

Small bowel neuroendocrine tumors (NETs) rarely present as obscure overt gastrointestinal bleeding (OGIB), causing significant diagnostic challenges. We report a 56-year-old man who presented with OGIB that required approximately 50 units of packed red blood cells during a 6-week hospitalization. Multimodal evaluation, including computed tomography angiography, tagged red blood cell scan, video capsule endoscopy, computed tomography enterography, provocative mesenteric angiography, balloon enteroscopy, and octreotide scan, failed to identify a source. Exploratory laparotomy identified 3 mid-ileal nodules with histopathology revealing well-differentiated grade 1 neuroendocrine tumors. Bleeding resolved following resection. This case highlights the diagnostic and therapeutic role of surgical exploration in refractory OGIB.

## INTRODUCTION

Obscure overt gastrointestinal bleeding (OGIB) accounts for approximately 5% of gastrointestinal bleeding cases and commonly originates from the small bowel.^1^ Malignancy is an uncommon but clinically important cause of gastrointestinal bleeding. Among primary small bowel malignancies, neuroendocrine tumors (NETs) are the most common, although their incidence is only 1.05 per 100,000 persons.^2^

Gastrointestinal bleeding is a rare manifestation of small bowel NETs and may be difficult to diagnose. While advances in endoscopic and radiologic imaging have improved the evaluation of small bowel bleeding, establishing the underlying etiology remains difficult in some patients. We present a patient with transfusion-dependent OGIB caused by multifocal grade 1 ileal NETs that were diagnosed only after surgical resection.

## CASE REPORT

A 56-year-old man with a history of end-stage renal disease on hemodialysis and hypertension presented to the emergency department with gastrointestinal bleeding. He had 2 days of melena as well as 1 episode of bright red blood per rectum. His initial hemoglobin was 8.1 g/dL, with a baseline hemoglobin of 9–10 g/dL, and decreased to 6.2 g/dL within 24 hours.

Initial computed tomography (CT) angiography did not demonstrate active bleeding. A tagged red blood cell nuclear scan showed active bleeding localized to the small bowel (Figure 1). Repeat computed tomography angiography was again unable to identify a bleeding source. Esophagogastroduodenoscopy (EGD) and push enteroscopy were unremarkable, while colonoscopy demonstrated dark red blood and clots throughout the colon extending to the ileocecal valve without an identifiable source of bleeding, suggesting an obscure overt small bowel bleed (Figure 2). Video capsule endoscopy identified an irregular, mass-like small bowel fold associated with intermittent oozing of fresh blood (Figure 3). The location of this abnormality within the small bowel could not be reliably determined. CT enterography did not demonstrate any discrete bowel wall thickening or mass lesion. Superior mesenteric artery angiography with provocative measures, including intravenous heparin, tissue plasminogen activator, and intra-arterial nitroglycerin, failed to demonstrate active extravasation. Retrograde single-balloon enteroscopy revealed heme-filled debris throughout the distal small bowel without identification of a discrete bleeding source (Figure 4). Octreotide scan was negative.

**Figure 1.**
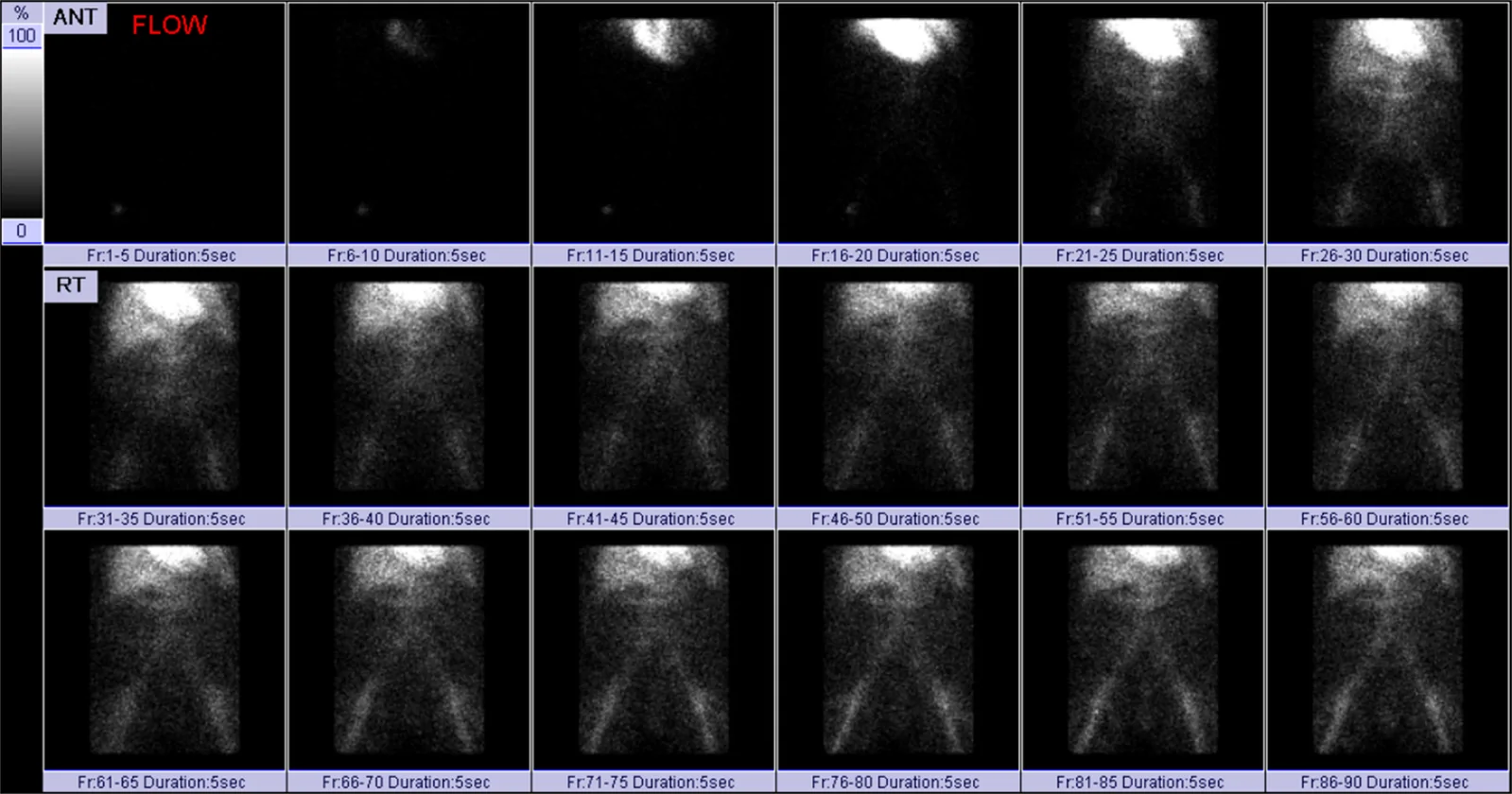
Tagged red blood cell nuclear scan showing active gastrointestinal bleeding within the small bowel.

**Figure 2.**
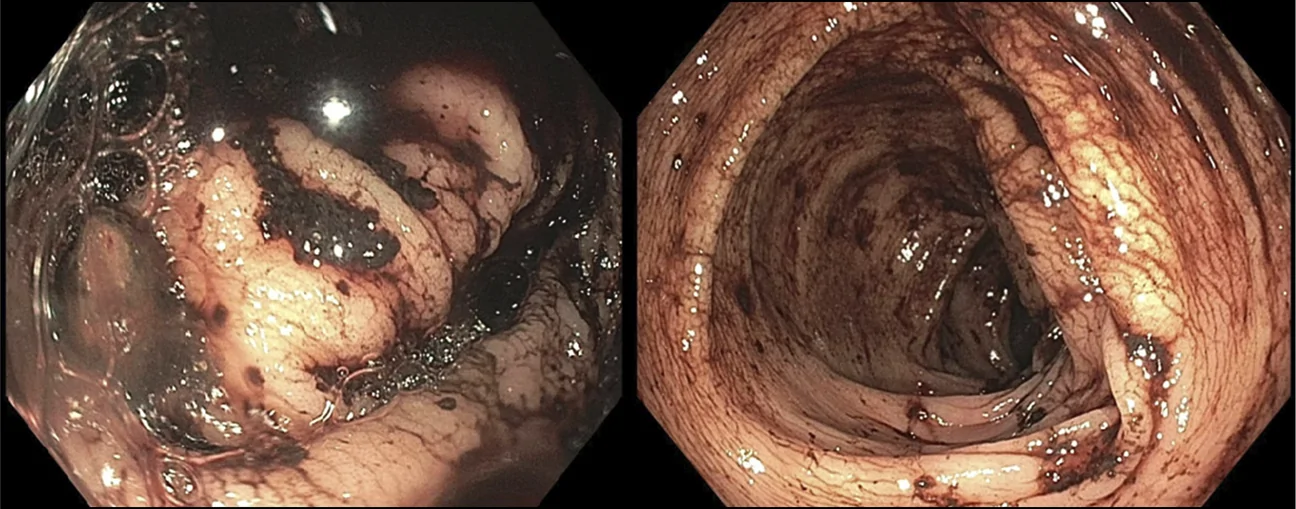
Colonoscopies showing (left) dark red blood in the cecum and (right) dark fluid and stool throughout the colon.

**Figure 3.**
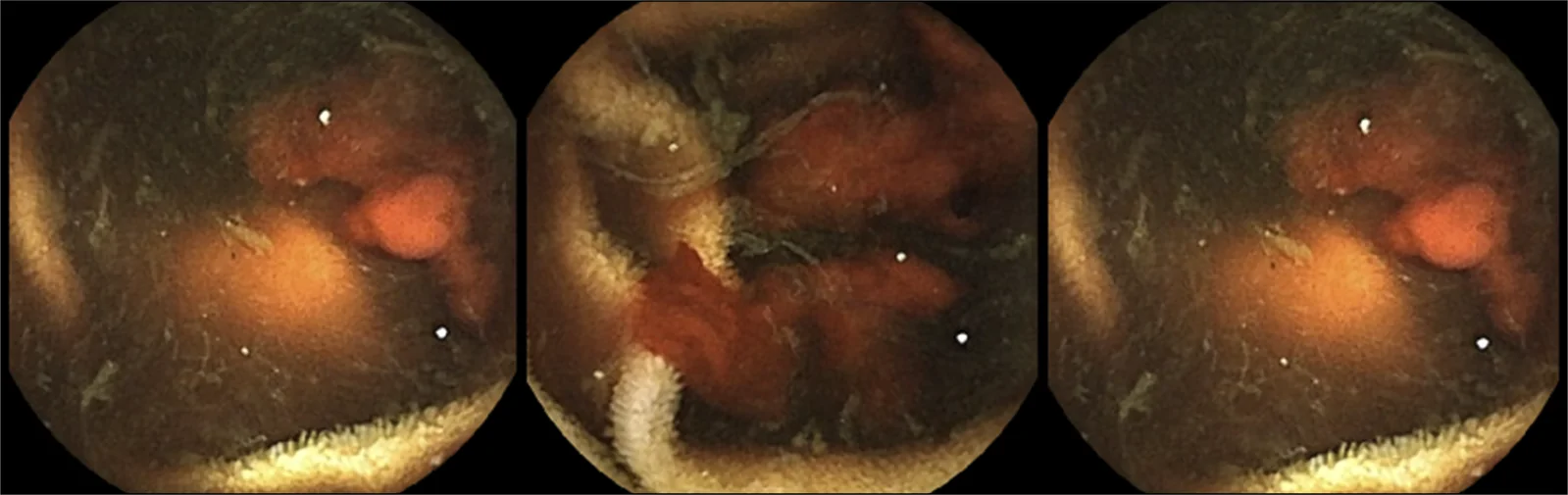
Video capsule endoscopy showing irregular, mass-like small bowel fold with intermittent oozing red blood.

**Figure 4.**
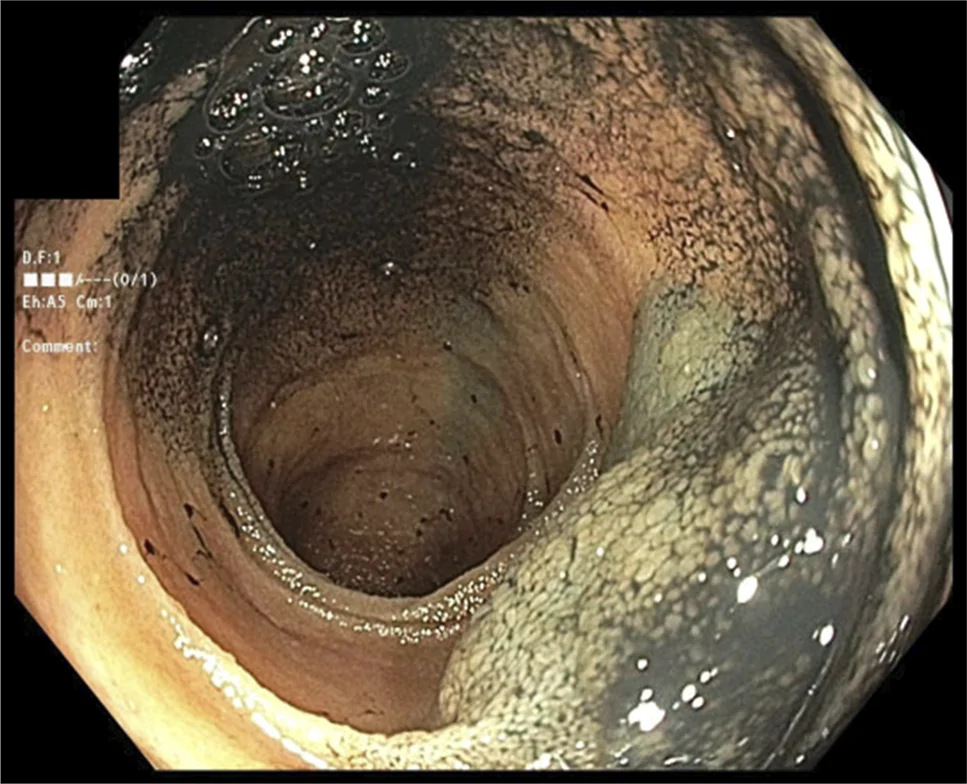
Retrograde single balloon enteroscopy showing heme-filled debris and dark, liquid stool within the distal small bowel.

Despite extensive evaluation, bleeding persisted, and packed red blood cells were transfused when the hemoglobin fell below 7.0 g/dL. He required approximately 50 units of packed red blood cells over a 6-week hospitalization but remained hemodynamically stable with a mean arterial pressure ≥ 65 mm Hg. Because of his operative risk related to his end-stage renal disease and continued hemodynamic stability, additional attempts at endoscopic and radiographic localization were pursued before exploratory laparotomy with intraoperative esophagogastroduodenoscopy.

At surgery, the patient underwent small bowel resection. Three small nodules were identified in the mid-ileum, approximately 8–10 cm apart. Pathologic examination demonstrated 3 multifocal, well-differentiated grade 1 primary ileal NETs, the largest measuring 1.0 cm. The tumors exhibited a mitotic rate of fewer than 2 mitoses per 2 mm^2^ and a Ki-67 proliferation index of < 3%. One tumor invaded the muscularis propria (pT2), while the remaining 2 tumors were confined to the submucosa (pT1). All resection margins were negative for disease. No lymphovascular invasion or mesenteric masses were identified. The final pathologic stage was pT2 (1 tumor) and pT1 (2 tumors) according to the American Joint Committee on Cancer 8th edition staging system. Following surgical resection, the patient had complete resolution of gastrointestinal bleeding.

## DISCUSSION

Small bowel NETs are often characterized as indolent neoplasms. Most patients present with abdominal pain, bowel obstruction, or metastatic disease, while overt gastrointestinal bleeding is uncommon.^3,4^ This case illustrates a discordance between tumor biology and clinical presentation. In a study conducted by Araujo et al,^5^ patients with grade 1 NETs experienced no disease progression or disease-related deaths during follow-up. Similarly, Jann et al^6^ reported a 100% 5-year survival rate among patients with grade 1 midgut NETs. Despite their low-grade histology, the tumors in our patient caused life-threatening gastrointestinal hemorrhage, demonstrating that tumor grade does not necessarily predict the severity of bleeding.

Another important aspect of this case is the inability of advanced diagnostic modalities to establish a diagnosis. Current algorithms for OGIB emphasize the use of capsule endoscopy, cross-sectional enterography, balloon-assisted enteroscopy, angiography, and functional imaging.^7,8^ Although capsule endoscopy suggested a focal abnormality, multimodal evaluation neither reliably localized the lesion nor established a diagnosis.

The diagnostic challenge of small bowel NETs stems from several inherent pathologic and anatomical features. These tumors are characteristically submucosal and may not deform the overlying mucosa sufficiently to be recognized on endoscopy or produce discrete bowel wall thickening on cross-sectional imaging. Their small size and intermittent bleeding pattern also limit the yield of angiography, CT enterography, and scintigraphy, all of which depend on either sufficient mass effect or active hemorrhage during the study.^9–11^ Multifocality is present in up to 50% of ileal NETs, adding additional complexity, as synchronous lesions may be missed on capsule endoscopy or enteroscopy.^12^ Octreotide scintigraphy has limited sensitivity for lesions under 1 cm.^13^ A study by Keck et al^14^ found that in 155 cases of NETS with metastases, somatostatin receptor scintigraphy was only able to identify 88 of the 155 primary lesions in the gastrointestinal tract or pancreas. Together, these features explain why even a multimodal evaluation failed to identify the ileal NETs in our patient.

Review of the literature demonstrates that gastrointestinal bleeding from small bowel NETs is uncommon. In most reported cases, diagnosis is established through identification of a lesion on endoscopic evaluation, angiography, enterography, or surgical exploration prompted by radiographic findings or metastatic disease. Few published reports describe similarly transfusion-dependent bleeding from small bowel NETs diagnosed only after surgical exploration.

Ultimately, surgical intervention provided both definitive diagnosis and control of the bleeding. This case highlights that in carefully selected patients with persistent transfusion-dependent OGIB, surgical exploration should be considered when repeated endoscopic and radiographic investigations remain nondiagnostic.

## DISCLOSURES

Author contributions: J. Mo conceptualized the clinical data, conducted the literature review, and wrote the initial manuscript draft. S. Suresh managed the patient, provided clinical guidance, contributed to manuscript revision, and approved the final version. J. Mo is the article guarantor.

Financial disclosure: None to report.

Previous presentation: Poster presentation at American College of Gastroenterology (ACG) 2026 Annual Scientific Meeting on October 13, 2026 in Nashville, TN.

Informed consent was obtained from the patient's next of kin.

## ABBREVIATIONS:

CT: computed tomography
EGD: esophagogastroduodenoscopy
NETs: neuroendocrine tumors
OGIB: overt gastrointestinal bleeding
